# Enhancing Botulinum Toxin Injection Precision: The Efficacy of a Single Cadaveric Ultrasound Training Intervention for Improved Anatomical Localization

**DOI:** 10.3390/toxins16070304

**Published:** 2024-07-02

**Authors:** Camille Heslot, Omar Khan, Alexis Schnitzler, Chloe Haldane, Romain David, Rajiv Reebye

**Affiliations:** 1 Division of Physical Medicine and Rehabilitation, University of British Columbia, Vancouver, BC V6T 1Z4, Canada; 2 Canadian Advances in Neuro-Orthopedics for Spasticity Congress (CANOSC), Kingston, ON K7K 1Z6, Canada; 3 Faculty of Medicine, Paris Cité University, 75006 Paris, France; 4 Department of Physical Medicine and Rehabilitation, GH St Louis Lariboisière F. Widal, 75010 Paris, France; 5 Hotel Dieu Shaver Health and Rehabilitation Centre, St. Catharines, ON L2T 4C2, Canada; 6 PRISMATICS Lab (Predictive Research in Spine/Neuromodulation Management and Thoracic Innovation/Cardiac Surgery), Poitiers University Hospital, 86000 Poitiers, France; 7 Department of Physical Medicine and Rehabilitation, Poitiers University Hospital, 86000 Poitiers, France

**Keywords:** ultrasound-guided, chemodenervation, botulinum toxin, muscle spasticity

## Abstract

Ultrasound guidance can enhance existing landmark-based injection methods, even through a brief and single exposure during a cadaveric training course. A total of twelve participants were enrolled in this training program, comprising nine physical medicine and rehabilitation specialists, one pediatrician, and two physician assistants. For each participant, one upper-limb muscle and one lower-limb muscle were randomly chosen from the preselected muscle group. Subsequently, participants were tasked with injecting both of their chosen cadaveric muscles with 1 mL of acrylic paint using a manual needle palpation technique, relying solely on their knowledge of anatomic landmarks. Participants then underwent a personalized, one-to-one ultrasound teaching session, lasting approximately five minutes, conducted by two highly experienced instructors. Following this instructive phase, participants were tasked with a second round of injections, targeting the same two muscles in the lower and upper limbs. However, this time, the injections were performed using anatomical landmarks and ultrasound guidance. To facilitate differentiation from the initial injections, a distinct color of acrylic paint was employed. When employing the anatomical landmark-based approach, the overall success rate for injections was 67%, with 16 out of 24 targeted muscles accurately injected. With the incorporation of ultrasound guidance, the success rate was 92%, precisely targeting 22 out of the 24 muscles under examination. There was an improvement in injection accuracy achievable through the integration of ultrasound guidance, even with minimal training exposure. Our single cadaveric ultra-sound training program contributes valuable insights to the utilization of ultrasound for anatomy training to help optimize the targeting of BoNT-A.

## 1. Introduction

Focal spasticity, characterized by muscle overactivity and increased tone, often presents a significant challenge in achieving precise and effective treatment following central nervous system injuries [1,2]. Among the various therapeutic approaches available, botulinum toxin type A (BoNT-A) injections have emerged as a first-line treatment for modulating specific muscle overactivity [3,4,5].

While BoNT-A injections have proven to be an invaluable tool in alleviating spasticity-related impairments, their efficacy is closely tied to optimal dose and precise targeting of affected muscles [6,7]. Indeed, achieving optimal treatment outcomes hinges upon the accurate identification and injection of the spastic muscle(s) [8,9,10,11].

Appropriate targeting of the spastic muscle(s) is of paramount importance, as the accuracy of injection location directly impacts treatment outcomes [12,13]. Traditional injection techniques based on anatomical landmarks have long served as the foundation of BoNT-A therapy. However, emerging evidence suggests that substantial enhancements in accuracy and efficacy can be achieved through the integration of ultrasound guidance [14,15,16,17,18,19,20]. The application of ultrasound in BoNT-A injections has garnered increasing attention, leading to a growing demand for ultrasound-guided BoNT-A injection courses [21,22,23].

As a result of problems with accuracy in using surface anatomy for muscles localization, there has been an increase in ultrasound education for targeting spastic muscles internationally and numerous courses have been developed to meet this need [12,17,24,25,26,27,28].

It has been shown that cadaveric-based anatomy learning is important to reinforce a clinician’s understanding of key muscles that are targeted for treatment with BoNT-A and can be complemented with ultrasound anatomy teaching [29,30,31,32].

In addition, incorporating the use of ultrasound to visualize muscle structures can help complement and improve anatomy knowledge and can in turn help improve procedural skills [33,34,35].

Our course included a component of a short ultrasound identification teaching (5 min) of muscles that were initially targeted using the traditional identification of muscles using surface-anatomy landmarks and from textbook landmarks that could help in improving the targeting of the muscles. 

This paper, therefore, aims to show that integrating ultrasound guidance in a cadaveric spasticity course can significantly enhance existing landmark-based injection methods, even through a brief and single exposure during a cadaveric training course. By investigating the value of brief training in echo-guided BoNT-A injections, we hope to contribute valuable insights to the evolving landscape of BoNT-A therapy for focal spasticity management.

Within this context, our data collected were for quality assurance of our course but also to shed light on the comparison between traditional cadaveric-anatomy-based injection techniques and ultrasound-guided approaches for anatomical teaching, with a particular focus on commonly treated spastic muscles. 

## 2. Results

All participants in the study successfully completed the comprehensive training course. The subsequent cadaveric dissections offered valuable insights into the precision of injection techniques. 

When employing the anatomical landmark-based approach, the overall success rate for injections was determined to be 67%, with 16 out of 24 targeted muscles accurately injected. Within the upper limb, 8 out of 12 muscles were correctly targeted using the anatomical landmark technique. These successfully injected upper-limb muscles included triceps brachii, biceps brachii, brachialis, brachioradialis, pronator teres, flexor carpi radialis, flexor digitorum superficialis, and adductor pollicis. Notably, four upper-limb muscles—flexor carpi ulnaris, flexor digitorum profundus, flexor pollicis longus, and pronator quadratus—were missed when relying solely on anatomical landmarks. In the lower limb, the anatomical landmark technique achieved a success rate of 8 out of 12 muscles. These correctly injected lower-limb muscles included rectus femoris, vastus intermedius, iliopsoas, adductor magnus, gracilis, soleus, flexor hallucis longus, and tibialis posterior. Four lower-limb muscles, including adductor longus, medial gastrocnemius, lateral gastrocnemius, and flexor digitorum longus were missed when using the anatomic landmark technique. 

With the incorporation of ultrasound guidance, there was an increase in the overall injection success rate, which surged to 92%, precisely targeting 22 out of the 24 muscles under examination. During this phase, only one upper-limb muscle, the adductor pollicis, and one lower-limb muscle, the adductor magnus, were not accurately injected (Table 1).

Significantly, when employing ultrasound guidance, the eight muscles that were initially missed with the anatomical landmark technique were accurately targeted. Conversely, the two muscles missed with ultrasound guidance were successfully injected using the anatomical landmark technique.

Statistical paired analyses, conducted to assess the impact of ultrasound guidance on injection accuracy, revealed a highly significant effect (*p* < 0.001). These findings underscore the substantial enhancement in precision and efficacy afforded by ultrasound guidance during BoNT-A injections for focal spasticity management in both the upper and lower limbs.

## 3. Discussion

Our findings reveal a notable improvement in injection accuracy when utilizing ultrasound guidance in addition to traditional manual palpation-based techniques. This higher success rate with ultrasound-guided injections underscores the substantial impact of incorporating ultrasound technology into the BoNT-A injection process when designing education curriculum and for the management of focal spasticity.

**Figure 1:**
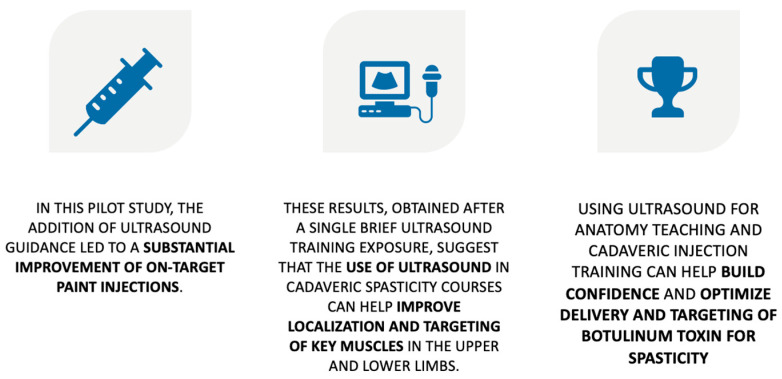
Key points of this study.

The clinical implications of these results are significant (Figure 1). Firstly, the incorporation of ultrasound for anatomical teaching is valuable to enhance the identification of key muscles for BoNT-A injections. Secondly, the enhanced accuracy achieved through ultrasound-guided injections can translate directly into improved patient outcomes. Accurately targeting the intended muscles is paramount in optimizing the therapeutic effects of BoNT-A, ultimately leading to improved spasticity management. Patients may experience reduced muscle hypertonia, decreased pain, improved motor function, and an enhanced quality of life. Furthermore, accurate muscle targeting can also reduce the rate of adverse effects, such as unwanted muscle weakness or pain from inadvertent injection of neighboring neurovascular structures.

Moreover, improved accuracy can potentially reduce the need for higher BoNT-A dosages, minimizing the risk of adverse effects and optimizing the utilization of this valuable therapeutic resource. Clinicians can also expect enhanced predictability in treatment outcomes, allowing for more tailored and effective treatment plans.

Our findings align with previous studies that have supported the use of ultrasound in guiding BoNT-A injections [36,37,38]. The literature consistently suggests that ultrasound guidance offers substantial advantages in terms of accuracy and precision. This includes studies highlighting the utility of ultrasound in various clinical contexts, such as musculoskeletal interventions [39,40,41,42].

There are many instances in which experienced clinicians rely on the anatomic descriptions found in textbooks for electromyographers to landmark the muscle to be injected when using guidance such as EMG for the BoNT-A injection technique [43,44]. Haig et al. revealed that the accuracy of blind needle placement in a cadaveric study using fine wire insertion according to muscle landmarks by Delagi and Perotto, Gierienger by seasoned Electromyographers was low. In this study, 36 different muscles in 10 cadaveric lower limbs were targeted with a fine wire using textbook landmarks of Delagi, Perotto, and Geiringer resulting in 263 targeted muscles. An anatomist blinded to the intended location dissected and recorded the muscles and other tissues that the wire pierced or passed nearby. The results showed that only 45% of the wire tip was in the intended muscle. Specific muscle accuracy was highly variable from 0% in 12 deep hip muscles to 100% in the vastus medialis [45].

An observational study by Henzel et al., using surface anatomy landmarks (based on Delagi and Perotto and anatomical published landmarks by Bickerton) to inject 18 patients with upper extremity flexor spasticity with BoNT-A and then using ultrasound for visualization of the muscle intended to be injected revealed significant variability regarding the accuracy of BoNT-A injection using surface anatomy and landmark-based palpation compared to ultrasound. The Henzel et al. study highlighted that surface anatomy localization by itself may not be very accurate, and that ultrasound can be used to enhance muscle targeting [43,46,47].

While our results reinforce these previous findings, it is essential to note that our study specifically examined the impact of a single brief exposure to ultrasound guidance to reinforce anatomy localization within a cadaveric model. This approach complements anatomy teaching and can help correct misguidances associated with anatomical landmarking techniques. The trainees reported improved procedural confidence after the brief ultrasound guidance teaching compared to their first injection attempt using only anatomical landmark based injection. 

The success of anatomical landmarks in targeting the adductor pollicis and adductor magnus, despite the failing of ultrasound-guided injections, can be attributed to the unique challenges posed by small muscle targets and deep muscle targets using the out-of-plane technique in novice ultrasound injectors. The out-of-plane technique requires more practice for needle tip identification and tracking when injecting deeper seated muscles. 

Despite our positive results, several limitations must be acknowledged. Firstly, the order of injections, whether based solely on anatomic landmarks or a combination of anatomical landmarks and ultrasound, was not randomized. This potential source of bias should be carefully considered when interpreting the results. The order of muscle injections was chosen from a proximal to distal orientation in order to avoid/limit disruption of freshly injected acrylic paint by subsequent injections by reducing repeat ultrasound probe pressure over injected muscles.

Furthermore, our study’s relatively small sample size, consisting of 12 participants, and the fact that each muscle was injected only twice by a single participant, warrant caution when generalizing our findings. Additionally, the cadaveric model, while valuable for controlled experiments, may not fully replicate the clinical environment.

Another notable limitation of our study is the retrospective data collection method employed. This retrospective approach prevented us from determining which specific trainees faced challenges in injection accuracy and subsequently analyzing potential contributing factors such as their level of experience or specialty. While this limitation restricts our ability to offer deeper insights into the individual performance of trainees, it is important to highlight that despite the majority of trainees having little to no prior experience in ultrasound-guided injections, the overall success rate in ultrasound-guided injections remained notably high. This finding emphasizes the feasibility and potential for rapid proficiency in ultrasound-guided injection techniques, highlighting the promise of integrating this technology into the clinical practice of various healthcare professionals, even those who are relatively new to the methodology.

Further research with larger cohorts and clinical studies involving live patients will be instrumental in corroborating and expanding upon our observations.

It is important to note that our course utilizing the short brief ultrasound exposure does not imply that the clinicians become experts in utilizing the ultrasound on a single exposure. It takes a significant amount of training, volume of patients seen, and attending more formalized courses to become proficient in the use of ultrasound for targeting botulinum toxin into spastic muscles. Our course demonstrates that a single ultrasound exposure can help reinforce anatomy teaching and can be incorporated into existing cadaveric-based anatomy teaching to help optimize the clinician’s confidence in visualizing the muscle to be injected.

## 4. Conclusions

The use of ultrasound in cadaveric-based BoNT-A intervention training demonstrates the substantial improvement in injection accuracy achievable through the integration of ultrasound guidance, even with minimal training exposure. 

The combination of ultrasound within a cadaveric course should be considered when developing an educational curriculum at the residency and for clinicians attending cadaveric-based spasticity injection courses.

The ability to visualize muscle and neurovascular structures in real time can help the clinician better understand the “whole picture” rather than just looking at the target. The ultrasound image can allow the clinician to look beyond the target (i.e., looking beyond the muscle to be injected and also assessing other adjacent muscles and neurovascular and bony structures) and this may allow better visualization and a better appreciation of a patient’s anatomy. The techniques employed by this study, including a brief exposure to focused, high-level ultrasound training to repeat a muscle target injection with acrylic paint followed by cadaveric dissection of the injected limbs allowed the trainees to analyze and compare the advantages and disadvantages of anatomical landmarking versus ultrasound-guided injections. While the study has its limitations, it contributes valuable insights to the evolving landscape of BoNT-A therapy for focal spasticity management, setting the stage for the enhancement of curriculum design and for future investigations and advancements in this field.

## 5. Materials and Methods

### 5.1. Study Participants and Settings

A comprehensive two-day training course was held at a university cadaveric lab offering formal anatomy teaching, didactic instruction, and hands-on ultrasound scanning using cadaveric specimens. There were 2 physicians instructors experienced in ultrasonography and one anatomist for cadaver dissection.

Pre-COVID, this was a yearly course conducted since 2016, and primary data for program evaluation from the last course in 2019 was collected as part of the course design and then used as secondary data for quality assurance of the cadaveric teaching and ultrasound course. 

This course was tailored explicitly to early-career clinicians who had already completed their post-graduate training and had some experience in botulinum toxin type A (BoNT-A) injections, with less than five years of clinical practice. A total of twelve participants were enrolled in this training program, comprising nine physical medicine and rehabilitation (PMR) specialists, one pediatrician, and two physician assistants, all in the first five years of their professional practice.

Whole-body cadaveric specimen tissue was prepared using Surgical Reality Fluid (Trinity Fluids, LLC., Harsens Island, MI, USA) to facilitate tissue storage and longevity while preserving the natural characteristics required to achieve limb pliability and maintain soft tissue sonographic characteristics [48]. 

Acrylic paints by ARTIST’S LOFT™ (Michaels store, Oakville, ON, Canada https://canada.michaels.com (accessed on 1 December 2023)) were utilized for intra-muscular injection in the cadaveric specimen. After testing several variations of India ink with thickening agents such as cornstarch and injectable hardening agents, it was established by one of the authors (O.K.) that acrylic paint maintained the most ideal characteristics for ease of injection, essentially unlimited injection time after syringe preparation and good injection localization within cadaveric tissue. It was also very cost-effective and consistent between all injectate colors.

The ultrasound machines used were Esaote MyLab 7 equipped with a 13–3 Hz linear array transducer, (Esaote; location: Fishers, IN, USA) and a 13–3 Hz linear array transducer, (Esaote; location: Fishers, IN, USA), with MSK (B-mode) and a setting and depth of 4 cm for the upper limb and 5 cm for the lower limb. Focal zone and gain settings were established by the training instructors for image optimization.

### 5.2. Study Design

#### 5.2.1. Anatomic Landmark Injections

The muscles commonly targeted for BoNT-A injection were preselected by two experienced training instructors. For the upper limb, the study focused on the following 12 muscles: triceps brachii, biceps brachii, brachialis, brachioradialis, pronator teres, flexor carpi radialis, flexor carpi ulnaris, flexor digitorum superficialis, flexor digitorum profundus, flexor pollicis longus, pronator quadratus and adductor pollicis. In the lower limb, the selected muscles included adductor longus, adductor magnus, rectus femoris, vastus intermedius, iliopsoas, gracilis, soleus, medial gastrocnemius, lateral gastrocnemius, flexor digitorum longus, flexor hallucis longus, and tibialis posterior. 

For each participant, one upper-limb muscle and one lower-limb muscle were randomly chosen from the preselected muscle group. Subsequently, participants were tasked with injecting both of their chosen cadaveric muscles with 1 mL of acrylic paint and 2-inch 21G needles using a manual needle palpation technique, relying solely on their knowledge of anatomic landmarks. The ultrasound-guided injection technique used was out of plane for all the muscles.

#### 5.2.2. Ultra-Sound Individualized Training

Subsequently, each participant underwent a personalized, one-to-one ultrasound teaching session, lasting approximately five minutes, conducted by two highly experienced instructors. During this session, the instructors guided the participants in utilizing ultrasound in addition to their knowledge of anatomical landmarks to precisely identify the previously targeted muscles in both the lower and upper limbs.

#### 5.2.3. Ultra-Sound Guided Injections

Following this instructive phase, participants were tasked with a second round of injections using in-plane ultrasound-guided injections, targeting the same two muscles in the lower and upper limbs. However, this time, the injections were performed using anatomical landmarks and ultrasound guidance. To facilitate differentiation from the initial injections, a distinct color of acrylic paint was employed. The interval between the ultrasound individualized training and the ultrasound-guided injections was set at 30 min, allowing participants to apply the newly acquired ultrasound skills promptly.

#### 5.2.4. Analysis of the Injection’s Accuracy

To rigorously assess the precision and accuracy of the injection techniques employed, an experienced anatomist with expertise in musculoskeletal anatomy conducted meticulous cadaveric dissections. These dissections were carried out blindly, without prior knowledge of the specific injection techniques used but also blinded with regards to the meaning of ink color (i.e., the color-coding of “first” vs. “second” injection) and the intended target muscle. These dissections were undertaken with the specific objective of discerning the exact locations of acrylic paint placement resulting from the anatomic landmark-based injection approach and from the ultrasound-guided injection technique.

### 5.3. Statistical Analysis

All statistical analyses were performed using the Jamovi software tool (version 2.3.28). We conducted paired statistical analysis, specifically McNemar’s Test, to compare participant performance before and after individualized ultrasound training. The significance level was set at *p* = 0.05 to determine the statistical significance of any observed changes.

### 5.4. Ethics

Participation in the cadaveric teaching program was based on the university’s anatomical lab agreement to consent to the anatomy lab’s ethical standards. The authors state that every effort was made to follow all local and international ethical guidelines and laws that pertain to the use of human cadaveric donors in anatomical research.

Quality assurance and quality improvement (QA/QI) studies, program evaluation activities, performance reviews, or testing within normal educational requirements when used exclusively for assessment, management, or improvement purposes, do not constitute research under the TCPS 2 (2018) and do not fall under the scope of REB of the University of British Columbia review (https://ethics.gc.ca/eng/tcps2-eptc2_2018_chapter2-chapitre2.html#5, accessed on 1 December 2023).

## Figures and Tables

**Table 1:** Results of the injection accuracy in the targeted muscles. HIT: properly targeted injection, MISS: Unproperly targeted injection.

Target Muscle	Anatomical Guidance	Ultrasound Guidance
**Upper limb**		
triceps brachii	HIT	HIT
biceps brachii	HIT	HIT
brachialis	HIT	HIT
brachioradialis	HIT	HIT
pronator teres	HIT	HIT
flexor carpi radialis	HIT	HIT
flexor carpi ulnaris	MISS	HIT
flexor digitorum superficialis	HIT	HIT
flexor digitorum profundus	MISS	HIT
flexor pollicis longus	MISS	HIT
pronator quadratus	MISS	HIT
adductor pollicis	HIT	MISS
**Lower Limb**		
adductor longus	MISS	HIT
adductor magnus	HIT	MISS
rectus femoris	HIT	HIT
vastus intermedius	HIT	HIT
iliopsoas	HIT	HIT
gracilis	HIT	HIT
soleus	HIT	HIT
medial gastrocnemius	MISS	HIT
lateral gastrocnemius	MISS	HIT
flexor digitorum longus	MISS	HIT
flexor hallucis longus	HIT	HIT
tibialis posterior	HIT	HIT

## Data Availability

Data are not publicly available but can be sent by authors on reasonable requests.
